# Darifenacin Versus Parasacral Transcutaneous Electric Nerve stimulation for overactive bladder syndrome in patients infected with Human T-Lymphotropic Virus 1 – randomized open clinical trial

**DOI:** 10.1590/S1677-5538.IBJU.2024.0567

**Published:** 2025-04-15

**Authors:** Tatiane Souza Soares de Oliveira, José Abraão C., Cassius José Vitor Oliveira, Néviton M. Castro, Edgar M. Carvalho

**Affiliations:** 1 Unidade Multidisciplinar do Departamento de Fisioterapia do Complexo Hospitalar Professor Edgard Santos da Universidade Federal da Bahia Salvador Bahia Brasil Unidade Multidisciplinar do Departamento de Fisioterapia do Complexo Hospitalar Professor Edgard Santos da Universidade Federal da Bahia (UFBA) Salvador, Bahia, Brasil;; 2 Divisão de Imunologia do Complexo Hospitalar Professor Edgard Santos da Universidade Federal da Bahia Salvador Bahia Brasil Divisão de Imunologia do Complexo Hospitalar Professor Edgard Santos da Universidade Federal da Bahia (UFBA) Salvador, Bahia, Brasil;; 3 Divisão de Urologia do Complexo Hospitalar Professor Edgard Santos da Universidade Federal da Bahia Salvador Bahia Brasil Divisão de Urologia do Complexo Hospitalar Professor Edgard Santos da Universidade Federal da Bahia (UFBA) Salvador, Bahia, Brasil;; 4 Instituto Gonçalo Moniz Laboratório de Pesquisa Clínica Fiocruz Bahia Brasil Laboratório de Pesquisa Clínica (LAPEC), Instituto Gonçalo Moniz, Fiocruz-Bahia, Brasil;; 5 Instituto Nacional de Ciência e Tecnologia em Doenças Tropicais Ministério de Inovação Científica e Tecnológica do Brasil Brasil Instituto Nacional de Ciência e Tecnologia em Doenças Tropicais (INCT-DT), Ministério de Inovação Científica e Tecnológica do Brasil

**Keywords:** Urinary Bladder, Overactive, Electric Stimulation, Randomized Controlled Trials as Topic

## Abstract

**Purpose:**

To evaluate the efficacy of parasacral transcutaneous electric nerve stimulation (PTENS), in comparison to darifenacin for the reduction of OAB symptoms in patients infected with HTLV-1.

**Materials and Methods:**

This proof-of-concept randomized clinical trial was carried out at the HTLV-1 Outpatient Clinic of the University Hospital. Participants included 42 HTLV-1 infected subjects with symptoms OAB. The OAB symptoms score questionnaire (OABSS) was applied before and after treatment to evaluate each group: group1-received darifenacin and group 2-treated with PTENS. Random sequences and statistical analysis were generated by SPSS statistical package, version 27 (IBM Inc™).

**Results:**

There was no difference between groups regarding demographic, socio-economic and clinical characteristics. The initial median and interquartile (IQR) range of OABSS were 11.2 (9.5 - 14.0) in G1 and 10.7 (8.0 - 12.7) in G2. There was a reduction in the frequency, nocturia and urgency in both groups. However, 5 (23.8%) of the patients in the group treated with darifenacin abandoned the therapy, while only 1 patient (4,8%) stopped PTENS.

**Conclusions:**

Both protocols used in this study were effective in treating OAB syndrome and reducing OABSS. However, therapy abandonment and adverse events were more frequent in the darifenacin group compared to the PTENS group.

## INTRODUCTION

Overactive bladder (OAB) is defined by the International Continency Society (ICS) as urinary urgency, usually accompanied by increased daytime frequency and/or nocturia, with urinary incontinence (OAB-wet) or without (OAB-dry), in the absence of urinary tract infection or other detectable disease (1). The main urologic dysfunction in HTLV-1 infection is OAB, observed in about 77.8% of the patients with associated myelopathy and 58.7% of infected subjects without motor disability (2). Moreover, it is responsible for adversely affecting daily activities, social, emotional, and economic relationships with a negative impact on quality of life when compared to other urinary dysfunctions (3, 4).

Currently, drug treatment for OAB in this population is based mainly on anticholinergic agents. The efficacy of treatment with antimuscarinics and anticholinergics drugs range from 50% to 70% (5). However, low adherence due to high incidence of adverse reactions, such as dry mouth, decrease of intestinal peristalsis, blurred vision, urinary retention and gastro-esophageal reflux leads to treatment interruption in approximately 30% of individuals (5–7). In addition to these adverse effects, patients with HTLV-1 suffer from motor disability, mental disorders, and pain. Patients with urinary symptoms, therefore, need new therapies that do not cause additional complaints or further compromise quality of life. Physiotherapy and more selective medications that do not cross the blood-brain barrier would add advantages to the care of these patients.

Electrical stimulation has been proven to improve urinary function in the bladder filling phase and is used in patients with neurogenic and non-neurogenic OAB (8–10). Although there is no consensus about the ideal type of electrostimulation parameters such as duration of the treatment, treatment location, or impulse length, positive outcomes have been documented. These outcomes are achieved because of the balancing of adrenergic and cholinergic neurotransmitters through electrical stimulation (11, 12).

The low rate of adherence to treatment with anticholinergic drugs and the improvement mediated by electrical nerve stimulation in patients with urinary dysfunction due to HTLV-1 (8) gives support to the evaluation of this form of therapy for HTLV-1 infected subjects with OAB symptoms. We hypothesize that PTENS may improve OAB symptoms with a lower incidence of adverse events compared to anticholinergic therapy. The objective of this study is to evaluate the efficacy of PTENS in reducing OAB symptoms in patients infected with HTLV-1 in comparison to darifenacin, once there is no study in the literature comparing the efficacy of these treatments in this population.

## MATERIALS AND METHODS

### Type of Study and Case Definition

This is a proof-of-concept randomized clinical trial carried out at the HTLV-1 Outpatient Clinic of a tertiary hospital, during the period from March 2022 to December 2023. Patients infected with HTLV-1 and presenting OAB according to ICS were invited to participate in the study. All patients presenting urinary symptoms of OAB had urodynamic studies. HTLV-1 infection was documented by ELISA (Cambridge Biotech, Worcester, MA) and confirmed by the Western-blot (HTLV Blot 2.4, Genelabs, Science Park Drive, Singapore) tests. Inclusion criteria were patients with HTLV-1 associated OAB older than 18 years. OAB was defined according to International Continence Society criteria (1). Exclusion criteria were HTLV-1 infected subjects with HTLV-1 associated myelopathy (HAM) (13), patients with previous history of stroke, Parkinson's disease, HIV, HBV, HCV, tertiary syphilis or HTLV - 2 infection, benign prostatic hyperplasia (BPH), pacemaker implantation, untreated genitourinary tract infection, glaucoma, and those who refused to participate in the study or sign the informed consent. This study was approved by the research ethics committee of the University Hospital on August 19, 2022 - CAAE: 59171022.9.0000.0049.

Subjects were invited to participate in the study according inclusion criteria by the urology staff. Those who accepted were randomly allocated to two groups (G1 and G2) by the free randomization website (14). After allocation, patients underwent an evaluation that included socio-demographic variables and the assessment of OAB symptoms using the OABSS questionnaire (15). Group1 was treated with darifenacin, and group 2 with the PTENS protocol. Evaluations were performed twice: before and after treatment. Both groups were evaluated on the first day of care. In Group 1, the re-evaluation took place on Day 60 of the darifenacin treatment, and in Group 2, after 20 sessions of PTENS, approximately 60 days after treatment initiation.

No patients received guidance on lifestyle changes and behavioral therapies and were off anticholinergics for at least 2 months.

### Drug Treatment Protocol:

The anticholinergic darifenacin was used in a single dose of 15mg/day for 2 months. The drug was provided free and delivered to patients by our research nurse team in an outpatient setting until the end of the study.

### PTENS Protocol:

The treatment consisted of PTENS with the Neurodyn™ Portable TENS FES (16) neuromuscular stimulation device in an outpatient clinic. Two self-adhesive electrodes (5x9cm) were used, positioned one in each gluteal region, below the posterior superior iliac spine to apply low-frequency biphasic current with 10 Hz. Pulse duration of 0.5 milliseconds was applied for 40 minutes with continuous stimulation, 3 times a week, for 20 sessions.

Therapeutic failure was defined by a symptom reduction lower than 20% of initial urinary complaints or discontinuation of therapy due to possible adverse reactions. Patients who failed treatment with PTENS were offered to receive darifenacin and vice versa.

## Statistical Analysis

Descriptive analysis measured mean and standard deviation (SD) for quantitative variables, and frequency (percentage) for qualitative variables. The normal distribution of data was evaluated using the Kolmogorov–Smirnov test. The exact Fisher test was used to compare the proportions. The Wilcoxon test was applied to investigate the response to treatments in both groups.

## RESULTS

**Figure 1:**
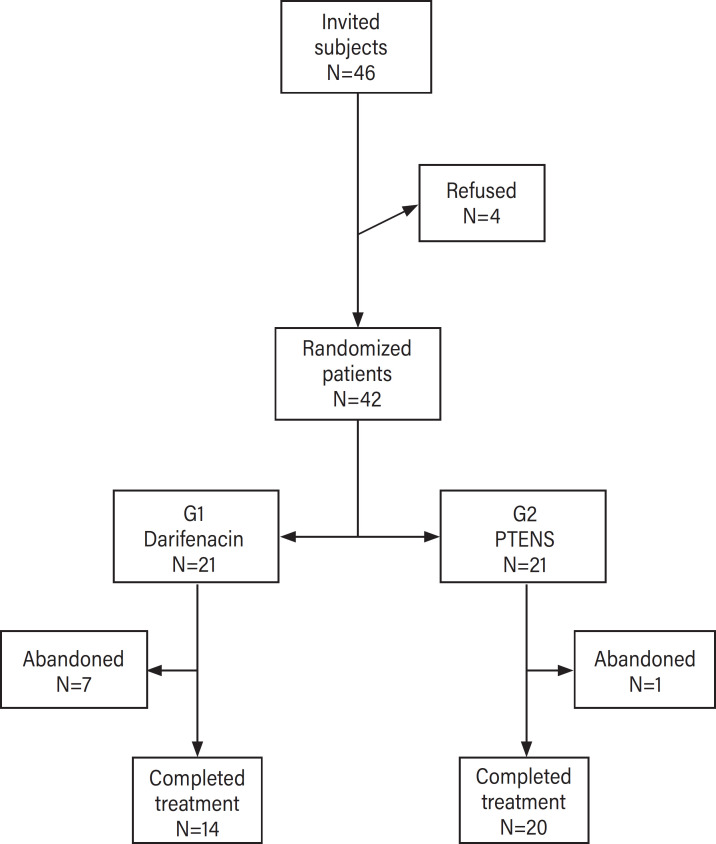
Flow of patients in the trial.

A total of 1,270 HTLV-1 infected subjects are being followed in the cohort study at the HTLV-1 Outpatient Clinic of the University Hospital, and 46 of them meeting the eligibility criteria for the study were invited to participate. One refused to participate after knowing the side effects of the medication and 3 refused physiotherapeutic treatment due to the inability to attend. Forty-two patients agreed to participate and were randomized to G1 or G2 (Figure-1).

The demographic, socio-economic, and general characteristics of the two groups are presented on Table-1. There was no difference regarding age, gender, income, education level, and OABSS. There was a predominance of mixed-skin color people in both groups, most people were Black.

**Table 1:** Demographic, Socio-economic and Clinical Characteristics of HTLV-1 Infected Subjects.

		G1 Darifenacin N=21	G2 PTENS N=21	P value
		N (%)	N (%)	
**Age, Years (M ± SD)**		57.5(10.4)	63.7(9.7)	0.869^a^
**Female Gender**		18(85.7)	20(95.2)	0.293^b^
**Monthly income**				
	<1 minimum wage	5(23.8)	7(33.3)	0.729^b^
	≥1 minimum wage	16(76.2)	14(66.7)	
**Education**				
	Illiterate	2(9.5)	1(4.8)	0.751^b^
	Elementary School	10(47.6)	12(57.1)	
	High school	9(42.9)	8(38.1)	
	Skin color			0,035^b^
	White	1(4.8)	0(0)	
	Brown	11(52.4)	4(19)	
	Black	9(42.9)	17(81)	
**OABSS**		11,19	10,67	0.629c
**Median (IQR)**		(9.5 – 14.0)	(8.0 – 12.7)	

HTLV-1 = Human T-Lymphotropic Virus 1;

a Student t test;

b Chi-square; P < 0.05;

c ANOVA.

The frequency of bladder symptoms and the OABSS in the two groups are shown on Table-2. All symptoms were present in both groups before treatment and improved significantly afterward. Urinary frequency and incontinence issues improved in response to the treatments, with a reduction from 76% of patients to 42.9% in G1, and from 61.9% to 30% in G2. About 50% of patients became continent in both groups. Regarding urgency, 42.9% of patients in G1 and 35% in G2 no longer presented urgency episodes. Nocturia was the symptom with less improvement, with 21.4% of patients not presenting this symptom in G1 and 20% in G2.

**Table 2:** Frequency Bladder Symptoms Before and After Treatments.

Variables		G1- Darifenacin		P value	G2- PTENS		P value
		N (%)			N (%)		
		Before	After		Before	After	
		N=21	N=14		N=21	N=20	
**Daily Frequency**				0.014			0.014
	≤ 7	5(23.8)	8(57.1)		8(38.1)	14(70.0)	
	8 - 14	15(71.2)	6(42.9)		13(61.9)	6(30.0)	
	≥ 15	1(4.8)	0(0.0)		0(0.0)	0(0.0)	
**Nocturia**				0.024			0.007
	0	0(0.0)	3(21.4)		0(0.0)	4(20.0)	
	1	1(4.8)	3(21,4)		2(9.5)	4(20.0)	
	2	3(14.6)	1(7.1)		4(19.0)	7(35.0)	
	≥ 3	17(81.0)	7(50.0)		15(71.4)	5(25.0)	
**Urgency**				0.010			0.009
	Never	0(0.0)	6(42.9)		0(0.0)	7(35.0)	
	< 1x/ week	0(0.0)	1(7.1)		2(9.5)	0(0.0)	
	≥ 1x/week	2(9.5)	1(7.1)		2(9.5)	3(15.0)	
	1x/day	2(9.5)	1(7.1)		0(0.0)	1(5.0)	
	2-4x/day	8(38.1)	1(7.1)		9(42.9)	3(15.0)	
	≥ 5x/day	9(42.9)	4(28.6)		8(38.1)	6(30.0)	
**Incontinence**				0.017			0.002
	Never	1(4.8)	7(50.0)		0(0.0)	10(50.0)	
	< 1x/ week	1(4.8)	0(0.0)		2(9.5)	1(5.0)	
	≥ 1x/week	5(23.8)	2(14.3)		5(23.8)	3(15.0)	
	1x/day	2(9.5)	1(7.1)		0(0.0)	2(10.0)	
	2-4x/day	6(28.6)	1(7.1)		8(38.1)	1(5.0)	
	≥ 5x/day	6(28.6)	3(21.4)		6(28.6)	3(15.0)	
**OABSS**		11.19	6.29	0.003	10.67	5.79	<0.001
**Median (IQR)**		(9.5 – 14.0)	(3.7 – 9.8)		(8.0 – 12.7)	(4.2 – 8.2)	
**Therapeutic** **Failure-dropout**			7 (33.3)			1(4.8)	0.04

PTENS = Parasacral Transcutaneous Electric Nerve Stimulation; OABSS = overactive bladder symptoms score; IQR = interquartile range.

There was a significant reduction in OABSS in both groups: from 11.2 to 6.3 in G1 (p = 0.003) and from 10.7 to 5.8 in G2 (p < 0.001). However, no significant difference was observed in the response between the groups (p = 0.79). Regarding the objective response to treatment, 8 of 14 and 11 of 20 patients showed a 50% or greater reduction in the OABSS score in the darifenacin and PTENS groups, respectively. However, the therapeutic failure rate was 33.3% in the darifenacin group and 4.8% in the PTENS group (p = 0.04). Side effects were observed in 15 (71.4%) of patients treated with darifenacin and in none of those treated with PTENS (Table-3). The most frequent causes for abandoning treatment with darifenacin reported by patients were constipation, anal bleeding, and dry mouth. Two patients who failed the initial treatment crossed over, one from each group, and both maintained their OABSS scores even after the second treatment.

**Table 3:** Frequency of Side Effects in HTLV-1 Infected Patients Treated with Darifenacin or Parasacral Transcutaneous Electric Nerve Stimulation.

		G1 Darifenacin	G2 PTENS
		N=21	N=21
Side effects		N (%)	N (%)
		15(71.4)	0
	Constipation	7(35.7)	0
	Anal bleedding	2(9.5)	0
	Sickness	1(4.7)	0
	Dry mouth	6(28.5)	0
	Vaginal dryness	1(4.7)	0
	Dysuria	1(4.7)	0
	Drownsiness	1(4.7)	0
	Epigastric pain	1(4.7)	0
	Dizziness	1(4.7)	0
	Tongue Bleeding	1(4.7)	0

## DISCUSSION

Urologic dysfunction in HTLV-1 infection is an important health problem not only due to its high prevalence, but also because it impairs the quality of life of infected subjects (3, 4). While OAB is the most important manifestation of urinary dysfunction, HTLV-1-infected subjects may also present with emptying dysfunction, including underactive bladder and the need for intermittent catheterization to empty the bladder (2). Conventional treatment for OAB is performed using drug therapy, bladder rehabilitation training, and surgery. The recommended first-line treatments for this dysfunction are behavioral interventions and pelvic floor muscle training (17). Bladder rehabilitation training can take a long time, and often the result is not always satisfactory. Surgery is invasive and dangerous due its associated complications. It is also unacceptable to some patients. PTENS is a therapeutic option and has been used for various disorders of the lower urinary tract. This technique is performed using surface electrodes in the sacral region (S2-S4) - segments with the parasympathetic innervation of the bladder, where a low-frequency current is applied in order to generate an inhibitory reflex to the detrusor muscle (18). Consequently, spinal and supraspinal pathways changes occur, modulating the activity of the postcentral gyrus (18). This leads to an increase in the maximum bladder cystometric capacity, including a reduction in all OABSS parameters: frequency, nocturia, urgency, and incontinence (19–21).

In the present study, we investigated the efficacy of PTENS and darifenacin in the treatment of OAB in patients with HTLV-1. Both treatments were effective in improving OAB, reinforcing the hypothesis that transcutaneous electric nerve stimulation of the parasacral region reduces the symptoms of OAB as much as darifenacin, but failure to therapy was higher in the darifenacin group than in patients treated with PTENS. The high rate of adverse reactions with darifenacin was the main cause for failure to therapy in patients treated with this drug. Recently it was shown that mirabegron was effective and had less adverse reactions than anticholinergic drugs in HTLV-1 infected individuals (22). However, as the cost of mirabegron is higher than anticholinergic agents, and HTLV-1 affects predominantly the poor population, darifenacin was used in the present study. Studies on PTENS have noted its ease of use, its non-invasiveness, and safety in treating OAB Syndrome. These qualities improve the acceptance of PTENS by patients (8). While PTENS therapy has demonstrated good results, it has been restricted to only women, young people and children with no neurogenic cause in most of them, evidencing the scarcity of studies in elderly individuals with neurogenic bladder, and in HTLV-1 infected subjects. This paper demonstrates good results with this technique in neurogenic bladder associated to HTLV-1, not previously tested. Based on this, the present protocol can corroborate the literature that affirms the role of transcutaneous electric nerve stimulation in the parasacral region to treat urinary symptoms.

During our study, we observed that most patients who had poorer results in both treatment groups reported symptoms of straining with sensation of incomplete bladder emptying, increased episodes of incontinence, which may be related to bladder underactivity. This population may have both a filling and emptying dysfunction, with emptying becoming more apparent after a possible improvement in bladder storage function. However, further studies with this focus are needed to confirm this statement.

The knowledge acquired in the present study helped clarify the role of electric stimulation in the treatment of OAB and enabled evidence-based recommendations on PTENS as part of first-line OAB syndrome treatment in HTLV-1 infected patients. However, we are aware our study has some limitations due a relatively small sample size, lack of a placebo group and lack of a cost-effectiveness analysis. Therefore, it is necessary to develop new studies that determine the most cost-effective way for choosing the technique most suitable for the patients infected by HTLV-1 presenting OAB. In the protocol of the present study, due to the nature of the therapy applied, it was not possible to blind the physiotherapist as well as the participants.

This study is significant for its originality as the first comparative analysis of anticholinergic treatment and electrostimulation in this population, supported by a randomized clinical trial. It demonstrates the non-inferiority of PTENS compared to anticholinergics and is particularly relevant considering emerging new perspectives for treating OAB in individuals infected with HTLV-1. Furthermore, it reinforces previous findings that PTENS for lower urinary tract dysfunction is safe, easy to administer, and well accepted by patients.

## CONCLUSIONS

Both protocols used in this study were effective in treating OAB syndrome and reducing OABSS. However, therapy abandonment and adverse events were more frequent in the darifenacin group compared to the PTENS group.

## ABBREVIATIONS

HTLV-1: = Human T-Lymphotropic Virus 1
HAM: = HTLV-1 associated myelopathy
IQR: = interquartile
ICS: = International Continency Society
OAB: = Overactive bladder
OABSS: = OAB symptoms score questionnaire
PTENS: = Parasacral transcutaneouselectric nerve stimulation
SD: = standard deviation
